# Supplementary material for: Parent’s Hesitation with COVID-19 Vaccinations in Infants and Children Aged 6 Months to 5 Years
Source: Vaccines (Basel). 2022 Oct 29;10(11):1828. doi: 10.3390/vaccines10111828 (PMC9693122; doi:10.3390/vaccines10111828)
Supplement: Supplementary file 1 [file vaccines-10-01828-s001.zip › vaccines-1950025-supplementary.pdf]

Attachment 1

Vaccine Hesitancy

Each family should only complete ONE questionnaire.

1. **What age most closely matches the age of your youngest child that you brought to the clinic today?**
   a. 6 months-11months
   b. 12 months-17 months
   c. 18 months-24months
   d. 25 months-36 months
   e. 3- less than 4 years old
   f. 4- less than 5 years old
2. **What is the sex of this child?**
   a. Male
   b. Female
3. **What is your relationship to this child?**
   a. Mother
   b. Father
   c. Guardian
   d. Family Member
   e. Other
4. **Does your child have a developmental problem or disability that would make it difficult for them to communicate? (i.e. language delay, autism, other)**
   a. No
   b. Yes
5. **What percentage of your family members have been vaccinated currently?**
   a. 0%
   b. 1-25%
   c. 26-50%
   d. 51-75%
   e. 75-100%
6. **What is your likelihood of vaccinating your child for COVID-19 at this or future visits?**
   a. Unlikely ever to vaccinate
   b. Unlikely but may consider in the future when I know more
   c. Unlikely today but will do it in the future from what I know now
   d. Likely would do it today if I had time so will schedule later
   e. Very likely to do it today if available
   f. My child has received the vaccine
7. **If you do have any hesitancy about vaccinating your child for COVID-19 what may be some of the reasons? (May select more than one)**
   a. Need more information
   b. Need to see more children immunized
   c. I don't think it is needed because my child does not have any risks
   d. I don't think the vaccine will work for my child
   e. I think the vaccine is more likely to harm my child than help them
   f. I don't trust the pharmaceutical companies
   g. I don't trust the establishment government/healthcare
   h. Would like more time to discuss with family
   i. Would like more time to discuss with my doctor
   j. I have religious reasons why I do not want to vaccinate
   k. I don't have any hesitancy about vaccinating
8. **For Individuals whose children have received at least one dose of a COVID-19 vaccine, but have not completed the dosage schedule, how likely are you to have your child receive subsequent doses?**
   a. Very likely for my child receive all recommended doses
   b. Likely for my child to receive the next recommended dose but am hesitant for my child to receive subsequent doses (if next dose does not complete the series)
   c. Hesitant for my child to receive any subsequent doses
   d. Not likely for my child to receive any more doses
   e. My child has not yet received any COVID-19 vaccine
   f. My child has received all recommended doses.
9. **Do you or anyone in your household have any of the following conditions? (select all that apply)**
   a. Cancer
   b. Chronic kidney disease
   c. Chronic obstructive pulmonary disease
   d. Heart conditions, such as heart failure, coronary artery disease or cardiomyopathies
   e. Obesity or severe obesity
   f. Sickle cell disease
   g. Type 2 diabetes mellitus
   h. Immunocompromised due to genetic factors or organ transplant
   i. Current smoker
   j. None of the above
10. **To your knowledge, do you have, or have you had COVID-19?**
   a. Yes
   b. No
   c. I don't know
11. **If your answer to #10 is "NO" skip this question. If "Yes" describe the level of care you received or are receiving:**
   a. Did not seek medical care
   b. Received medical care but was not hospitalized
   c. Was hospitalized
12. **Do you personally know anyone in your family, group of friends, or community networks who became seriously ill or died as a result of COVID-19?**
   a. Yes
   b. No

13. **How concerned are you about your child getting COVID-19?**

a. Not at all concerned
b. A little concerned
c. Moderately concerned
d. Very concerned

14. **How safe do you think a COVID-19 vaccine will be for your child?**

a. Not safe at all
b. A little safe
c. Moderately safe
e. Very safe

15**. What would motivate you to allow your child to be vaccinated? (select all that apply)**

a. Protect my child's health
b. Protect health of myself/friends/family
c. Protect health of child's friends and peers
d. Protect health of community
e. To get back to school/work
f. To resume social activities
g. To resume travel
h. Because others encouraged me to allow my child to be vaccinated
i. Other
j. Not sure

15. **Have you seen or heard any information about COVID-19 vaccines (e.g., on the news, on social media, or from friends and family) that you could not determine were true or false?**

a. Yes
b. No
c. Not sure

16. **How do you feel about the information on COVID-19 vaccines that you are getting?**

a. I'm not getting enough information
b. I'm getting enough information
c. I'm getting too much information

17. **Do you know where to get accurate, timely information about COVID-19 vaccines?**

a. Yes
b. No
c. Not sure

18. **Select you top 3 most trusted sources for information about COVID-19 vaccines:**

a. Centers for Disease Control and Prevention (CDC)
b. Employer
c. Family and Friends
d. Food and Drug Administration
e. Health Insurers
f. Hospital system websites (e.g., CHKD, Sentara)
g. Local health officials
h. News Sources (e.g., television, internet radio)
i. Nurses
j. Pharmacists
k. Primary care providers
l. Professional organization(s)
m. Religious leader(s)
n. State health departments
o. Online publishers of medical information (such as WebMD or Mayo Clinic)
p. Social medial (such as Facebook, Twitter, Instagram, WhatsApp, LinkedIn, or Tik-Tok)
q. Union leader(s)
r. Other

19. **Where do you live in Hampton Roads?**

a. Norfolk
b. Virginia Beach
c. Chesapeake
d. Portsmouth
e. Suffolk
f. Newport News
g. Hampton
h. Other

20. **What is your age**?

a. 18 to 24 years
b. 25 to 34 years
c. 35 to 44 years
d. 45 to 54 years
e. 55 to 64 years
f. Age 65 or older

21. **What level of schooling did you complete**?

a. Did not complete high-school
b. High School or GED
c. Some College including Business/Trade school
d. 2-year College
e. 4-year College

22. **What best describes your race/ethnicity**?

a. Caucasian-White
b. African-American- Black
c. Hispanic
d. Asian/Pacific Islander
e. Native American
f. Multi-racial
g. Other

23. **What was your total household income before taxes during the past 12 months?**

a. Less than $25,000
b. $25,000 to $34,999
c. $35,000 to $49,999
d. $50,000 to $74,999
e. $75,000 to $99,999
f. $100,000 to $149,999
g. $150,000 or more
h. Prefer not to answer
